# The extracellular volume status predicts body fluid response to SGLT2 inhibitor dapagliflozin in diabetic kidney disease

**DOI:** 10.1186/s13098-020-00545-z

**Published:** 2020-05-01

**Authors:** Ken Ohara, Takahiro Masuda, Masato Morinari, Mari Okada, Atsushi Miki, Saki Nakagawa, Takuya Murakami, Kentaro Oka, Maki Asakura, Yasuharu Miyazawa, Akito Maeshima, Tetsu Akimoto, Osamu Saito, Daisuke Nagata

**Affiliations:** 1 Division of Nephrology, Department of Internal Medicine, Jichi Medical University, 3311-1 Yakushiji, Shimotsuke, Tochigi 329-0498 Japan; 2 Department of Internal Medicine, Nasu Minami Hospital, Nasukarasuyama, Tochigi Japan

**Keywords:** SGLT2 inhibition, Bioimpedance analysis, Heart failure, BNP, Extracellular volume expansion, Hypovolemia, Furosemide, Loop diuretic, Tolvaptan, Vasopressin V2 receptor antagonist

## Abstract

**Background:**

Sodium–glucose cotransporter 2 (SGLT2) inhibitors are an antihyperglycemic drug with diuretic action. We recently reported that the SGLT2 inhibitor dapagliflozin ameliorates extracellular volume expansion with a mild increase in urine volume. However, the impact of the pretreatment extracellular volume status on the body fluid response to SGLT2 inhibitors remains unclear.

**Methods:**

Thirty-six diabetic kidney disease (DKD) patients were treated with dapagliflozin. The body fluid volume, including intracellular water (ICW), extracellular water (ECW) and total body water (TBW), were measured on baseline and day 7 using a bioimpedance analysis (BIA) device. The ECW/TBW and ECW were used as markers of the extracellular volume status. For a comparison, the extracellular volume status responses to loop diuretic furosemide (n = 16) and vasopressin V2 receptor antagonist tolvaptan (n = 13) were analyzed.

**Results:**

The body weight, brain natriuretic peptide and body fluid parameters measured by a BIA (ICW, ECW, TBW, and ECW/TBW) were significantly decreased for 1 week after dapagliflozin administration. The change in the ECW/TBW in the high-ECW/TBW group (over the median value of 0.413) was significantly higher than in the low-ECW/TBW group (− 2.1 ± 0.4 vs. − 0.5 ± 0.4%, *p *= 0.006). Only with dapagliflozin treatment (not furosemide or tolvaptan treatment) was the baseline ECW/TBW significantly correlated with the changes in the ECW/TBW (*r *= − 0.590, *p *< 0.001) and ECW (*r *= − 0.374, *p *= 0.025).

**Conclusions:**

The pretreatment extracellular volume status predicts the body fluid response to the SGLT2 inhibitor dapagliflozin in DKD patients. The diminished extracellular fluid reduction effect of dapagliflozin in patients without severe extracellular fluid retention may contribute to maintaining a suitable body fluid status.

## Background

Sodium–glucose cotransporter 2 (SGLT2) inhibitors are anti-diabetic drugs that increase the urinary glucose excretion by inhibiting SGLT2 in the early proximal tubule [1, 2]. Recent clinical trials have shown that SGLT2 inhibitors exhibit cardio-renal protective properties in patients with diabetes mellitus and diabetic kidney disease (DKD) [3, 4]. Because SGLT2 inhibitors induce notable benefits with regard to reducing hospitalization for heart failure, the importance of the diuretic action exerted by SGLT2 inhibition has been proposed [3]. In accordance, human and animal studies have shown that SGLT2 inhibitors increase the urine volume with an increase in sodium (Na^+^) and glucose excretion [5, 6], reflecting the fact that SGLT2 transports glucose together with Na^+^ (in a 1:1 ratio) [1].

We recently reported that the SGLT2 inhibitor dapagliflozin predominantly decreased the extracellular volume with a mild increase in the urine volume in DKD patients with fluid retention [7, 8]. Importantly, extracellular volume expansion, which is represented as an increased over-hydration index or the ratio of extracellular water (ECW) to total body water (TBW) (ECW/TBW), as measured by bioimpedance analysis (BIA) device, predicts high mortality and adverse cardio-renal outcomes in patients with chronic kidney disease (CKD) [9–12]. Thus, the reduction in the extracellular volume expansion through SGLT2 inhibition may be a promising cardio-renal protective mechanism in these patients.

On the other hand, we previously showed that the SGLT2 inhibitor ipragliflozin does not affect the body fluid volume (ECW, intracellular water [ICW] and TBW) in euvolemic animal models [6, 13], and recent meta-analyses did not demonstrate an increase in acute kidney injury with SGLT2 inhibitors [14, 15]. Taken together, these data suggest that SGLT2 inhibitors may have a protective effect against hypovolemia in addition to reducing the extracellular volume expansion. However, the homeostatic action of SGLT2 inhibitors on body fluid control has not yet been elucidated.

Based on these findings, we hypothesized that SGLT2 inhibitors decrease the body fluid volume in patients with fluid retention but show no such decrease in fluid volume for patients without fluid retention. We therefore examined the correlation between the pretreatment extracellular volume status and the body fluid response to the SGLT2 inhibitor dapagliflozin in DKD patients.

## Methods

### Patients

This study prospectively enrolled 36 DKD patients treated with dapagliflozin at Jichi Medical University (n = 13, Shimotsuke, Tochigi, Japan) and Nasu Minami Hospital (n = 23, Nasukarasuyama, Tochigi, Japan) between February 2016 and June 2019. DKD was defined as a combination of diabetes and an estimated glomerular filtration rate (eGFR) < 60 mL/min/1.73 m^2^ or the presence of proteinuria. In addition to the 14 patients enrolled in a previous study [7], another 22 patients with or without generalized edema were newly entered into this study. The average ECW/TBW, a marker of the extracellular volume status, in the newly entered patients was lower than that in the patients from the previous study (0.411 ± 0.005 vs. 0.421 ± 0.006, *p *= 0.082), as we aimed to examine the effect of dapagliflozin among a variety of extracellular fluid statuses. Among these 36 patients, 12 received a renal biopsy, and 7 had other types of renal diseases (glomerulonephritis, n = 4; minimal change, n = 3) in addition to DKD. The exclusion criteria were a history of renal replacement, current dialysis, type 1 diabetic mellitus or active malignancy.

The SGLT2 inhibitor dapagliflozin (5 mg/day) was administered in addition to the baseline medication. The patients with loop diuretic furosemide (n = 16, average dose 52.5 ± 11.5 mg/day) and vasopressin V2 receptor antagonist tolvaptan (n = 13, average dose 6.9 ± 0.4 mg/day), which partially include data from our previous study (2 newly added patients treated with furosemide and 1 patient treated with tolvaptan) [7], were compared with the enrolled patients treated with dapagliflozin. The treatment drug (dapagliflozin, furosemide or tolvaptan) was selected according to the judgment of the attending physicians. The main indication for administering tolvaptan was patients with diuretic-resistant heart failure for whom some diuretics had already been administered. Diuretics were not stopped at the initiation of the study, and no dose changes in the concomitant medications were allowed during the 7-day study period.

This study was conducted in accordance with the ethical principles of the Declaration of Helsinki. The study protocol and amendments were approved by the independent ethics committees of Jichi Medical University (Approval number: A17-014) and Nasu Minami Hospital (Approval number: 2016-03). All patients provided their written informed consent to participate in this study. The study was registered in the University Hospital Medical Information Network Clinical Trial Registry (UMIN-CTR) Clinical Trial (UMIN registration number: 000029863).

### Blood and urine sample collection

Blood samples and body weight data were collected before dosing on days 0 and 7 after the administration of dapagliflozin. The eGFR was calculated using the Modification of Diet in Renal Disease study equation coefficients modified for Japanese patients [16]. The stages of CKD were based on the NKF K/DOQI clinical practice guidelines [17]. The sample size with the data of baseline was as follows: blood pressure (BP), n = 25; plasma glucose, n = 23; brain natriuretic peptide (BNP), n = 27; serum albumin, n = 32; uric acid, n = 32; and proteinuria, n = 34. The sample size with the data of both days 0 and 7 was as follows: BP, n = 24; plasma glucose, n = 23; BNP, n = 19; serum albumin, n = 32; and uric acid, n = 32. Other parameters included all 36 patients’ data for baseline and day 7.

### Measurement of the fluid volume using a BIA

The body fluid volume was measured using a BIA device with eight tactile electrodes (InBody S10; InBody Japan Inc., Tokyo, Japan) before dosing at baseline and day 7 after dapagliflozin administration (3 h or later), similar to our previous studies [7, 8, 18–20]. The ICW, ECW, TBW (ICW + ECW) and ECW/TBW were calculated from the sum of each segment using the equations in the BIA software program. The InBody S10 user’s manual states that, “The normal range for ECW/TBW is considered 0.36–0.39. The ratio between 0.39 and 0.40 means ‘slight edema’, and over 0.40 means edema” [21]. Patients were classified into subgroups as follows: (i) according to the median value of ECW/TBW (low-ECW/TBW group [< 0.413] and high-ECW/TBW group [≥ 0.413]) (n = 36); and (ii) according to the median value of serum brain natriuretic peptide (BNP), a marker of extracellular fluid volume and heart failure (low-BNP group [< 95.7 pg/mL] and high-BNP group [≥ 95.7 pg/mL]) (n = 27) [22–24].

### Statistical analyses

The data were expressed as the mean ± standard error. Paired or unpaired t-tests were used to compare two variables, as appropriate. A one-way analysis of variance was used to compare the parameters with a normal distribution (dapagliflozin, furosemide and tolvaptan) among three groups. The Shapiro–Wilk test was used to check the normality of the distribution. Non-normally distributed data were presented as the median and interquartile range and were analyzed with Wilcoxon’s rank-sum test. The correlations among clinical parameters were analyzed using Pearson’s correlation (*r*). *p* values of < 0.05 were considered to indicate statistical significance. The statistical analyses were performed using the JMP Pro 14.2.0 software program (SAS Institute, Inc., Cary, NC, USA).

## Results

Table 1 shows the comparative parameters at baseline and 1 week after dapagliflozin treatment in the enrolled patients. Twenty-four (66.7%) patients used some form of diuretic (loop diuretic n = 24 [66.7%], mineralocorticoid receptor blocker n = 7 [19.4%], thiazide n = 3 [8.3%], tolvaptan n = 4 [11.1%]). The body weight, BMI, diastolic BP and levels of BNP, serum creatinine, uric acid and serum K^+^ significantly decreased from baseline to 1 week after dapagliflozin administration (Table 1). In contrast, the hemoglobin, hematocrit, serum albumin and eGFR significantly increased from the baseline. The plasma glucose level was not significantly changed after 1 week. All body fluid parameters measured by the BIA (ICW, ECW, TBW, and ECW/TBW) were significantly decreased from the baseline to 1 week later (Table 1). The HbA1c in the low-ECW/TBW group was significantly higher than in the high-ECW/TBW group (7.5% ± 0.2% vs. 6.8% ± 0.3%, *p *= 0.023).

**Table 1:** Comparative parameters at baseline and day 7 after dapagliflozin administration

Characteristics	Baseline (n = 36)	Day 7 (n = 36)	*p* value
Age (years)	68.5 ± 2.1	–	–
Male gender (%)	66.7	–	–
Body weight (kg)	68.6 ± 2.7	66.0 ± 2.5	< 0.001
BMI (kg/m^2^)	27.1 ± 0.7	26.1 ± 0.7	< 0.001
Systolic BP (mmHg)	139 ± 4	136 ± 5	0.210
Diastolic BP (mmHg)	76 ± 3	70 ± 3	0.012
Heart rate (beats/min)	75 ± 2	73 ± 3	0.450
Hemoglobin (g/dL)	11.1 (9.6–13.2)	11.3 (10.0–13.9)	0.002
Hematocrit (%)	32.9 (29.1–38.4)	34.3 (30.4–39.6)	0.009
Plasma glucose (mg/dL)	145 (116–206)	154 (115–184)	0.459
HbA1c (%)	7.2 ± 0.2	–	–
BNP (pg/mL)	91.6 (20.9–232.1)	60.8 (16.3–146.0)	< 0.001
Serum albumin (g/dL)	3.5 (2.8–4.0)	3.7 (3.0–4.0)	0.002
BUN (mg/dL)	26.2 (17.3–42.6)	24.8 (19.4–39.8)	0.183
Serum creatinine (mg/dL)	1.9 (1.4–2.3)	1.8 (1.3–2.6)	0.004
eGFR (mL/min/1.73 m^2^)	27.9 (19.2–42.0)	29.3 (17.9–39.6)	0.003
Uric acid (mg/dL)	6.6 ± 0.3	6.3 ± 0.2	0.031
Serum Na^+^ (mEq/L)	141 (138–142)	141 (137–143)	0.402
Serum K^+^ (mEq/L)	4.4 ± 0.1	4.2 ± 0.1	0.045
Proteinuria (g/day)^a^	1.3 (0.3–5.6)	–	
ICW (L)	21.8 ± 0.9	20.7 ± 0.8	< 0.001
ECW (L)	15.5 ± 0.7	14.3 ± 0.6	< 0.001
TBW (L)	37.2 ± 1.6	35.0 ± 1.4	< 0.001
ICW/TBW	0.585 ± 0.004	0.591 ± 0.003	< 0.001
ECW/TBW	0.415 ± 0.004	0.409 ± 0.003	< 0.001
Concomitant diuretics (%)	66.7		
Loop diuretics (%)	66.7	–	–
MR antagonist (%)	19.4	–	–
Thiazide diuretics (%)	8.3	–	–
Tolvaptan (%)	11.1	–	–

The sample size with the baseline data was as follows: BP, n = 25; plasma glucose, n = 23; BNP, n = 19; serum albumin, n = 32; uric acid, n = 32; and proteinuria, n = 34. Other parameters included all 36 patients’ data at baseline and day 7

Values are presented as the mean ± standard error or as the median (interquartile range), as appropriate

*BMI* body mass index, *BP* blood pressure, *BNP* brain natriuretic peptide, *BUN* blood urea nitrogen, *eGFR* estimated glomerular filtration rate, *ICW* intracellular water, *ECW* extracellular water, *TBW* total body water, *MR* mineralocorticoid receptor

^a^The urine protein–creatinine ratio was used in cases with only spot urine data available

Next, we examined the correlation between the baseline clinical parameters and ECW/TBW and the absolute change in the ECW/TBW, a marker of extracellular fluid volume [23]. The baseline ECW/TBW significantly correlated with the baseline GFR (*r* = − 0.593, *p* < 0.001), BNP (*r* = 0.495, *p* = 0.009) and HbA1c (*r* = − 0.607, *p* < 0.001). The hemoglobin, hematocrit, HbA1c and serum albumin were positively and significantly correlated with the absolute change in the ECW/TBW, whereas the BNP, uric acid, ECW, and ECW/TBW were negatively and significantly correlated with the absolute change in the ECW/TBW (Table 2). In contrast, the age, body weight, body mass index, systolic and diastolic BP, blood urea nitrogen, serum creatinine, serum Na^+^, serum K^+^, eGFR, ICW and TBW were not significantly correlated with the absolute change in the ECW/TBW (Table 2). The baseline ECW/TBW (0.424 ± 0.004 vs. 0.397 ± 0.003, *p* < 0.0001) and the absolute change in the ECW/TBW (− 0.009% ± 0.001% vs. 0.0003% ± 0.0020%, *p* = 0.001) were significantly higher in the patients receiving diuretic therapy than in those without any diuretic therapy.

**Table 2:** The correlation between the absolute change in the ECW/TBW and clinical parameters at baseline

Characteristics	*r* value	*p* value
Hemoglobin (g/dL)	0.466	0.006
Hematocrit (%)	0.420	0.011
HbA1c (%)	0.480	0.005
BNP (pg/mL)	− 0.598	0.001
Serum albumin (g/dL)	0.414	0.017
eGFR (mL/min/1.73 m^2^)	0.303	0.077
Uric acid (mg/dL)	− 0.398	0.024
ICW (L)	− 0.196	0.252
ECW (L)	− 0.383	0.021
ECW (L/1.73 m^2^)	− 0.538	< 0.001
TBW (L)	− 0.284	0.093
ECW/TBW	− 0.590	< 0.001

*BNP* brain natriuretic peptide, *eGFR* estimated glomerular filtration rate, *ICW* intracellular water, *ECW* extracellular water, *TBW* total body water

**Fig. 1:**
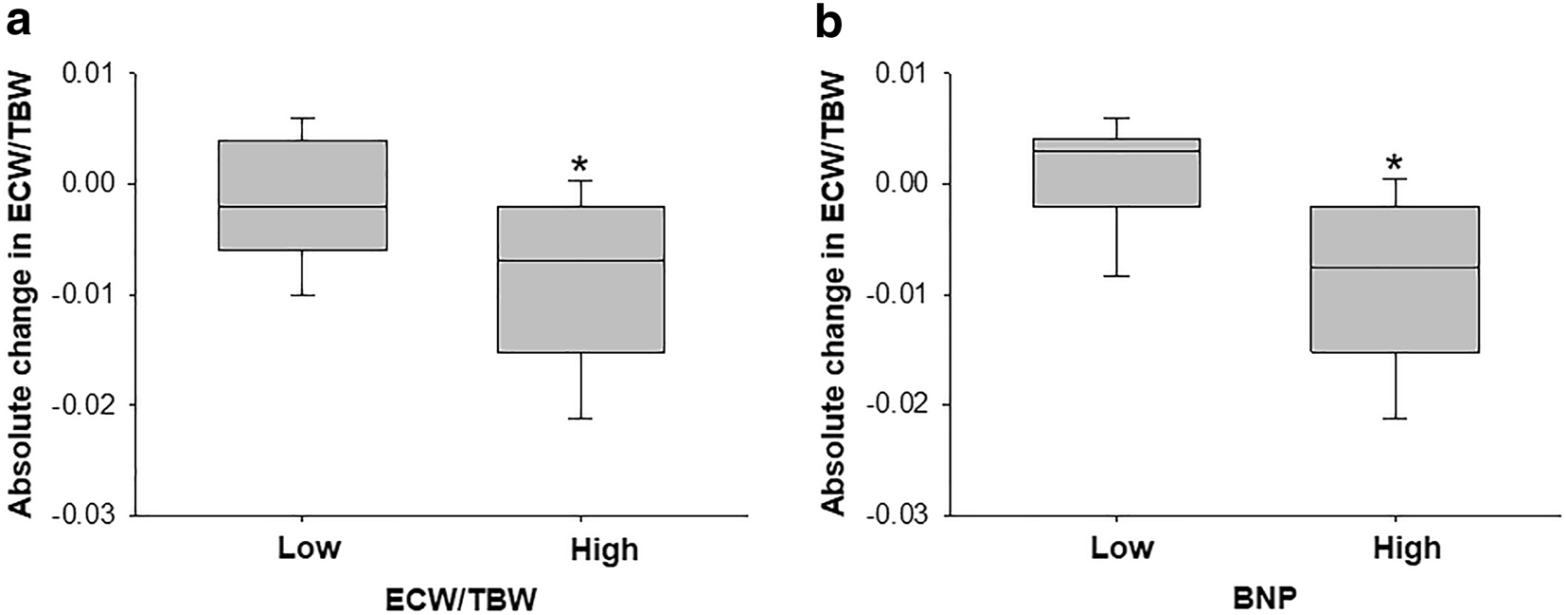
Absolute changes in the ECW/TBW depending on high vs. low ECW/TBW and the BNP at baseline. **a** The absolute change in the high-ECW/TBW group (over the median value of 0.413, n = 18) were significantly higher than those in the low-ECW/TBW group (less than the median value of 0.413, n = 18). **b** The absolute change in the high-BNP group (over the median value of 95.7 pg/mL, n = 14) were significantly higher than those in the low-BNP group (less than the median value of 95.7 pg/mL, n = 13). *ECW* extracellular water, *TBW* total body water. *p < 0.05 vs. low-ECW/TBW group or low-BNP group

We then compared the absolute change in the ECW/TBW with the two subgroups created based on the median value of the baseline ECW/TBW and BNP. The absolute change in the ECW/TBW in the high-ECW/TBW [n = 18] group was significantly higher than in the low-ECW/TBW group [n = 18] (− 2.1% ± 0.4% vs. − 0.5% ± 0.4%, *p *= 0.006) (Fig. 1a), and that in the high-BNP group [n = 14] was significantly higher than in the low-BNP group [n = 13] (− 2.39% ± 0.47% vs. − 0.03% ± 0.48%, *p *< 0.001) (Fig. 1b). In addition, the baseline ECW/TBW levels were divided into two groups with a cut-off value of 0.400, with edema being indicated if the value is over 0.400 based on the BIA device user manual [21]. The relative and absolute changes in the ECW/TBW and the relative and absolute changes in ECW in the low-ECW/TBW group (< 0.400) [n = 8] were all significantly lower than those in the high-ECW/TBW group (≥ 0.400) [n = 28] (Table 3).

**Table 3:** The comparison between the baseline ECW/TBW levels and the fluid response to dapagliflozin

Baseline ECW/TBW	Relative change in the ECW/TBW (%)	Absolute change in the ECW/TBW	Relative change in the ECW (%)	Absolute change in the ECW (L)
Low (< 0.400) [n = 8]	0.3 ± 0.6*	0.001 ± 0.003*	− 1.6 ± 2.8*	− 0.2 ± 0.5*
High (≥ 0.400) [n = 28]	− 1.7 ± 0.3	− 0.007 ± 0.001	− 8.1 ± 1.5	− 1.4 ± 0.3

Values are presented as the mean ± standard error

*ECW* extracellular water, *TBW* total body water

*p < 0.05 vs. ECW/TBW over 0.400 of each parameter

**Fig. 2:**
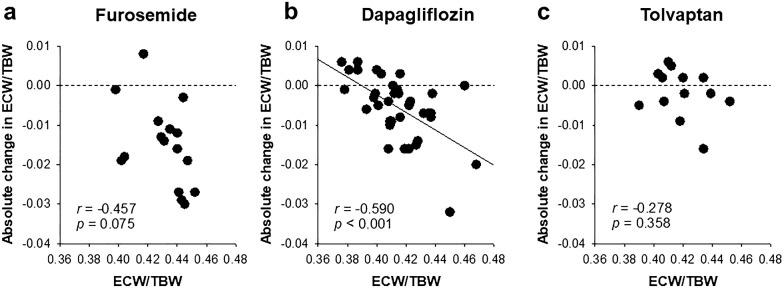
The correlation between baseline ECW/TBW and the absolute change in ECW/TBW. In the furosemide (**a**) and tolvaptan (**c**) groups, the ECW/TBW level was not significantly correlated, while in the Dapagliflozin group (**b**), it was negatively and significantly correlated. *ECW* extracellular water, *TBW* total body water

**Fig. 3:**
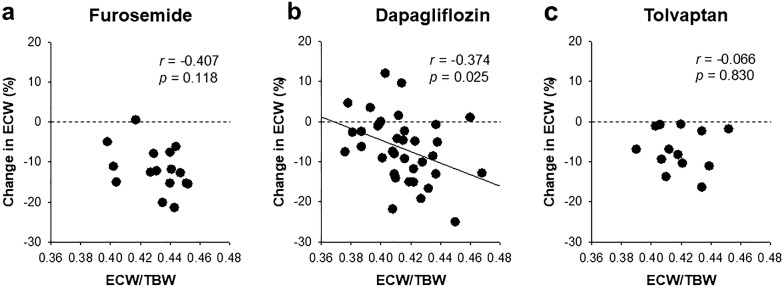
The correlation between baseline ECW/TBW and the change in ECW. In the furosemide (**a**) and tolvaptan (**c**) groups, the ECW/TBW level was not significantly correlated, while in the Dapagliflozin group (**b**), it was negatively and significantly correlated. *ECW* extracellular water, *TBW* total body water

Finally, we compared the fluid response to the SGLT2 inhibitor dapagliflozin, loop diuretic furosemide and vasopressin V2 receptor antagonist tolvaptan. The baseline body weight was similar among the three groups (Table 4). The baseline ECW/TBW was negatively and significantly correlated with the absolute change in the ECW/TBW only with dapagliflozin treatment (Fig. 2a–c). Similarly, the baseline ECW/TBW was negatively and significantly correlated with the change in the ECW only with dapagliflozin treatment (Fig. 3a–c). Furthermore, furosemide and tolvaptan deceased the absolute ECW values in almost all patients, regardless of differences in the baseline ECW/TBW values, although dapagliflozin induced a positive change in the absolute ECW values in several patients with a lower baseline ECW/TBW value (Fig. 3a–c).

**Table 4:** The comparison of the characteristics of patients treated with furosemide, dapagliflozin and tolvaptan (at baseline/at day 7)

Characteristics	Furosemide	Dapagliflozin	Tolvaptan	*p* value
Number	16	36	13	
Age (years)	70.1 ± 4.0	68.5 ± 2.1	72.9 ± 3.0	0.575
Male gender (%)	68.8	66.7	69.2	0.980
Body weight (kg) [baseline]	67.9 ± 3.0	69.0 ± 2.7	68.4 ± 4.9	0.988
BMI (kg/m^2^) [baseline]	26.0 ± 0.9	27.1 ± 0.8	27.3 ± 1.3	0.657
Diabetes kidney disease (%)	50.0	100	46.2	< 0.001
Systolic BP (mmHg) [baseline]	133 ± 7	138 ± 4	131 ± 5	0.621
Serum albumin (g/dL) [baseline]	1.9 (1.5–2.7)	3.5 (2.8–4.0)	2.7 (2.1–3.4)	< 0.001
eGFR (mL/min/1.73 m^2^) [baseline]	33.3 (13.3–60.3)	27.8 (19.2–42.0)	13.2 (9.4–21.5)	0.007
ECW/TBW [baseline]	0.431 ± 0.004	0.415 ± 0.004	0.419 ± 0.005	0.028
[day 7]	0.416 ± 0.004	0.409 ± 0.003	0.417 ± 0.005	0.224
Absolute change in ECW/TBW^a^	− 0.015 ± 0.002	− 0.005 ± 0.001	− 0.002 ± 0.002	0.001
Diuretics (%)	100	66.7	100	< 0.001

*BMI* body mass index, *BP* blood pressure, *eGFR* estimated glomerular filtration rate, *ICW* intracellular water, *ECW* extracellular water, TBW: total body water

^a^Day 7 vs. baseline. Values are presented as the mean ± standard error or as median (interquartile range)

## Discussion

This study shows that the fluid response to dapagliflozin varies depending on the baseline extracellular volume status in DKD patients. In more detail, dapagliflozin decreases the extracellular volume in patients with fluid retention, while not showing any such decrease in the extracellular volume in those without fluid retention. Until recently, several studies including ours have shown the effects of SGLT2 inhibitors on the body fluid status. In our previous studies of DKD patients with fluid retention, dapagliflozin predominantly decreased the ECW with a transient mild increase in urine volume [7, 8]. Recent studies of diabetes patients without fluid retention showed that the SGLT2 inhibitors transiently decreased the ECW or body fluid balance within 1 week, and returned to the initial value after that time [25, 26]. Although these data suggest the homeostatic action of SGTL2 inhibitors on the body fluid status, the separate examinations of fluid retention patients [7, 8] or non-fluid retention patients [25, 26] may not evaluate the effect of baseline extracellular fluid status on fluid response to SGLT2 inhibitors. In contrast, the present study, which included both fluid retention and non-fluid retention patients, clarifies the variable action of SGLT2 inhibitor dapagliflozin dependent on the baseline extracellular fluid status, and provides the usefulness of SGLT2 inhibitors for body fluid homeostasis.

Several mechanisms by which SGLT2 inhibitors maintain the homeostasis of body fluid volume have been proposed. Our recent study in non-diabetic euvolemic rats showed that SGLT2 inhibition with ipragliflozin induced a sustained glucose-driven diuretic and natriuretic tone, but homeostatic mechanisms—including compensatory increases in fluid and food intake—are activated to stabilize body fluid volume [6]. Furthermore, in our another study of diabetic rats, ipragliflozin induced a sustained osmotic diuresis with compensatory increases in vasopressin-induced solute-free water reabsorption to maintain body fluid volume, with an increase in renal aquaporin 2, which is expressed in the collecting duct [13]. The upregulation of the renin–angiotensin–aldosterone system (RAAS) may also be active counteracting mechanisms of fluid regulation after SGLT2 inhibition [5, 25, 27]. Schork et al. [25] reported that the transient decrease in the fluid status induced by SGLT2 inhibitors was accompanied by an increased activity of the RAAS after 30 days, which returned to the normal level after 6 months. Another promising mechanism is the compensatory Na^+^ and glucose reabsorption in the late proximal tubule. SGLT1, which is located in the distal parts of the proximal tubule and mediates the transport of glucose together with Na^+^ (in a 1:2 ratio) [28], is activated during SGLT2 inhibition [29]. Therefore, the compensatory increase in SGLT1-mediated transport during SGLT2 inhibition may act as a homeostatic mechanism for maintaining the body fluid volume through increasing the glucose and Na^+^ reabsorption in the late proximal tubule. Further studies with genetic or pharmacological SGLT1 inhibition during SGLT2 inhibition will be necessary in order to determine whether or not SGLT1 is involved in compensatory mechanisms of body fluid homeostasis during SGLT2 inhibition.

Is the homeostatic mechanism of body fluid volume by dapagliflozin due to a class effect of SGLT2 inhibitors? In our present and previous studies, neither dapagliflozin nor ipragliflozin decreased the extracellular fluid volume in patients without severe fluid retention (lower ECW/TBW patients) or in euvolemic rats [6, 13]. In contrast, dapagliflozin decreased the extracellular fluid volume in patients with fluid retention (higher ECW/TBW patients) [7, 8]. Recent studies involving another SGLT2 inhibitor (empagliflozin) also showed a maintenance effect of body fluid status in patients without fluid retention [25, 26]. Taken together, these findings suggest that SGLT2 inhibitors may have a homeostatic mechanism to normalize and maintain the body fluid status, and these effects may be a class effect of SGLT2 inhibitors.

In contrast to the SGLT2 inhibitor dapagliflozin, the body fluid responses to the loop diuretic furosemide and vasopressin V2 receptor antagonist tolvaptan did not clearly vary depending on the pretreatment extracellular volume status. The baseline ECW/TBW values in the furosemide and tolvaptan treatment groups were not significantly correlated with the changes in the ECW/TBW or ECW, respectively. Furthermore, furosemide and tolvaptan decreased the ECW in almost all patients, regardless of differences in the baseline ECW/TBW values, although dapagliflozin induced a positive change in the ECW in several patients with lower baseline ECW/TBW values. SGLT2 inhibitors suppress Na^+^ and glucose reabsorption in the early proximal tubule, which may activate sufficient and reasonable compensatory mechanisms in the more distant tubules to maintain body fluid balance. However, furosemide inhibits the Na^+^–K^+^–Cl^−^ cotransporter on the luminal site at the thick ascending limb of the loop of Henle, which is distal from the proximal tubule. Tolvaptan inhibits the absorption of water by selectively binding to the V2 receptor in the collecting duct, which is located on the most distal side of the tubule. When furosemide and tolvaptan inhibit the Na^+^ and fluid absorption at the thick ascending limbs or collecting duct, the compensatory Na^+^ and/or fluid reabsorption in more distant tubules may be weak or insufficient. Thus, unlike furosemide and tolvaptan, the SGLT2 inhibitor dapagliflozin is not merely a diuretic agent that generally uniformly reduces the body fluid volume regardless of the baseline fluid status; the fluid response to dapagliflozin varies depending on the baseline volume fluid status. In this regard, SGLT2 inhibitors exert different diuretic actions from loop diuretics and vasopressin V2 receptor antagonists. In addition, this pharmacological action of SGLT2 inhibitor may induce a novel clinical benefit that may support the safety (low risk of fluid shortage) of administering SGLT2 inhibitors to patients without fluid retention.

In the present study, dapagliflozin decreased the extracellular volume expansion in patients with higher baseline BNP levels, suggesting that SGLT2 inhibitors may ameliorate the extracellular volume expansion particularly effectively in patients with heart failure. A recent guideline of the European Society of Cardiology and European Association for the Study of Diabetes for diabetes, pre-diabetes and cardiovascular diseases recommends SGLT2 inhibitors be administered to reduce the risk of heart failure hospitalization [30]. The current findings may support the guideline recommendation in terms of ensuring effective body fluid control using SGLT2 inhibitors in patients with a high risk of developing heart failure hospitalization.

The baseline eGFR was weakly but positively correlated with the change in the ECW/TBW after the administration of dapagliflozin, suggesting that SGLT2 inhibitors can ameliorate body fluid retention even in patients with a reduced renal function. This result is quite different from the strength of glycemic control using SGLT2 inhibitors, in which the glucose-lowering effect decreases with a declining eGFR [31]. In the current study (average eGFR: 32.7 ± 3.1 mL/min/1.73 m^2^), dapagliflozin did not reduce the plasma glucose levels for 1 week. However, the change in the extracellular volume status induced by dapagliflozin was greater in patients with a lower eGFR than in those with higher values. Therefore, the strength of the diuretic action and the change in the extracellular fluid status induced by SGLT2 inhibitors may not be simply determined by the GFR-dependent filtered glucose and Na^+^ levels but may be mainly regulated by the compensatory reabsorption of Na^+^, glucose and fluid in more distant tubules, as we recently reported [13].

Several limitations associated with the present study warrant mention. First, this study had a relatively small sample size. Second, the relationship between the long-term body fluid response to SGLT2 inhibitors and the clinical outcomes is unclear, as this study had a short-term follow-up. Further follow-up studies are needed to evaluate this relationship. Third, the number of patients without fluid retention is small compared with the number of patients with fluid retention. Fourth, this was not a randomized control study, and a control group treated with vehicle was lacking.

## Conclusion

The pretreatment extracellular volume status predicts the body fluid response to the SGLT2 inhibitor dapagliflozin in DKD patients. These data suggest that the diminished extracellular fluid reduction effect of dapagliflozin in patients without extracellular fluid retention may contribute to maintaining a suitable body fluid status.

## Data Availability

The datasets are available from the corresponding author on reasonable request.

## Abbreviations

BIA: Bioimpedance analysis
BMI: Body mass index
BNP: Brain natriuretic peptide
BP: Blood pressure
BUN: Blood urea nitrogen
CKD: Chronic kidney disease
DKD: Diabetic kidney disease
ECW: Extracellular water
eGFR: Estimated glomerular filtration rate
ICW: Intracellular water
MR: Mineralo-corticoid receptor
SGLT2: Sodium–glucose cotransporter
TBW: Total body water
